# Therapeutic potential and mechanisms of mesenchymal stem cell-derived exosomes as bioactive materials in tendon–bone healing

**DOI:** 10.1186/s12951-023-01778-6

**Published:** 2023-01-16

**Authors:** Jiaxuan Zou, Weinan Yang, Wushi Cui, Congsun Li, Chiyuan Ma, Xiaoxiao Ji, Jianqiao Hong, Zihao Qu, Jing Chen, An Liu, Haobo Wu

**Affiliations:** 1 Department of Orthopedics, The Second Affiliated Hospital, Zhejiang University School of Medicine, Hangzhou, 310002 People’s Republic of China; 2 Orthopedics Research Institute of Zhejiang University, Hangzhou, 310002 People’s Republic of China; 3 Key Laboratory of Motor System Disease Research and Precision Therapy of Zhejiang Province, The Second Affiliated Hospital, Zhejiang University, Hangzhou, 310002 People’s Republic of China; 4 Clinical Research Center of Motor System Disease of Zhejiang Province, Hangzhou, 310002 People’s Republic of China; 5 The Second Hospital, Cheeloo College of Medicine, Shandong University, Jinan, 250033 People’s Republic of China

**Keywords:** Tendon–bone healing, Mesenchymal stem cells, Exosomes, Drug delivery, Nanocarriers, Biomaterials, Nanomedicine

## Abstract

Tendon–bone insertion (TBI) injuries, such as anterior cruciate ligament injury and rotator cuff injury, are the most common soft tissue injuries. In most situations, surgical tendon/ligament reconstruction is necessary for treating such injuries. However, a significant number of cases failed because healing of the enthesis occurs through scar tissue formation rather than the regeneration of transitional tissue. In recent years, the therapeutic potential of mesenchymal stem cells (MSCs) has been well documented in animal and clinical studies, such as chronic paraplegia, non-ischemic heart failure, and osteoarthritis of the knee. MSCs are multipotent stem cells, which have self-renewability and the ability to differentiate into a wide variety of cells such as chondrocytes, osteoblasts, and adipocytes. Numerous studies have suggested that MSCs could promote angiogenesis and cell proliferation, reduce inflammation, and produce a large number of bioactive molecules involved in the repair. These effects are likely mediated by the paracrine mechanisms of MSCs, particularly through the release of exosomes. Exosomes, nano-sized extracellular vesicles (EVs) with a lipid bilayer and a membrane structure, are naturally released by various cell types. They play an essential role in intercellular communication by transferring bioactive lipids, proteins, and nucleic acids, such as mRNAs and miRNAs, between cells to influence the physiological and pathological processes of recipient cells. Exosomes have been shown to facilitate tissue repair and regeneration. Herein, we discuss the prospective applications of MSC-derived exosomes in TBI injuries. We also review the roles of MSC–EVs and the underlying mechanisms of their effects on promoting tendon–bone healing. At last, we discuss the present challenges and future research directions.

**Graphical Abstract:**

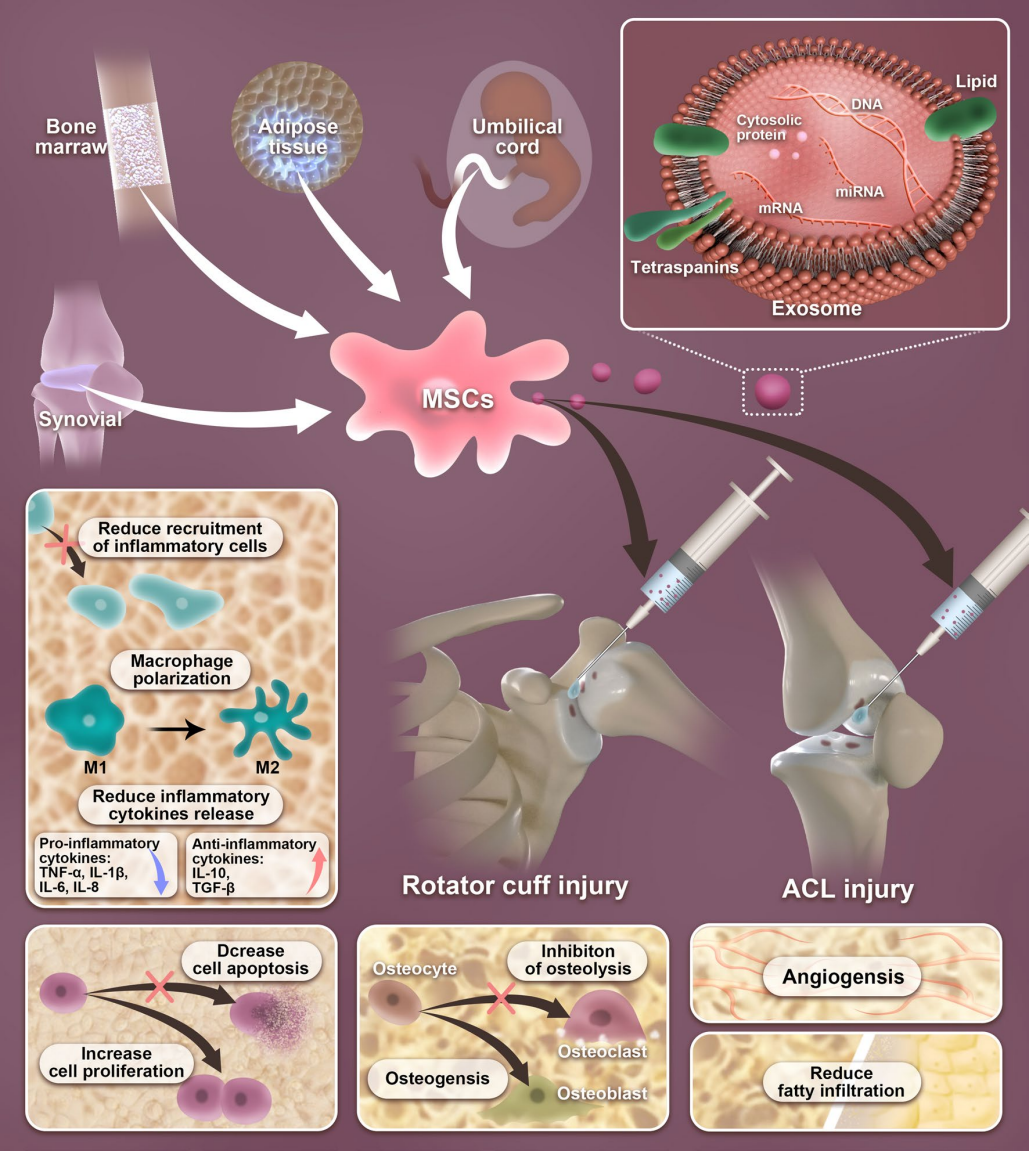

## Introduction

Tendon injuries are common musculoskeletal system disorders in orthopedic clinical practices that can cause excruciating pain and disability. Such injuries mostly occur during sports and routine daily activities [1]. The anterior cruciate ligament (ACL) and rotator cuff (RC) are two of the most commonly injured soft tissue structures. The interface where tendons and ligaments attach to the skeleton is referred to as the enthesis, which is the site of stress concentration in the region. Therefore, ACL and RC are prone to overuse injuries [2]. The incidence rate of RC injuries is very high in musculoskeletal diseases. Statistical data show that over 270,000 surgeries are performed annually in the United States to repair torn RC tendons [3]. In addition, approximately 200,000 patients per annum suffer from ACL injuries [4].

Clinically, surgical tendon/ligament reconstruction is required for treating RC and ACL enthesis injuries in most situations, which is accomplished by a tendon graft inserted into the bone tunnel to establish tendon-to-bone healing. However, the recurrent tear rate after the surgical intervention is high; for example, the re-tear rate for repaired RC was estimated to be as high as 94%, while the average ACL reconstruction failure rate was 11.7% [5, 6]. Sufficient tendon graft–bone healing depends on the degree of the bone ingrowth into the graft, and the regeneration of fibrocartilage zones is critical for satisfactory clinical outcomes of these injuries [7, 8]. The tendon–bone insertion (TBI), the transition between the soft tendinous tissue and the hard bony tissue, heals slowly as a result of hypovascularity, especially in the fibrocartilage zone. Thus, fibrocartilage healing occurs through scar tissue rich in type I collagen formation after an injury [5, 9, 10]. The fibrovascular scar with low biomechanical properties results in an inferior interface and increases the risk of injuries again because scar tissue lacks the native tendon–bone interface gradient structure and the alignment of collagen fiber [11]. To reduce the re-tear rate and achieve satisfactory surgical results, various approaches, such as using different suturing ways, interference screws, tissue engineering, and growth factors, have been applied to promote tendon–bone healing in animal models and patients. However, their efficacy is limited due to hypocellularity and poor vascularization of the tendon [12]. It is especially difficult for TBI regeneration when compared to bone-to-bone healing [13]. Consequently, fibrocartilage transition regeneration is currently limited and imposes a formidable challenge in TBI repair.

Recently, increasing studies have focused on mesenchymal stem cells (MSCs), which are multipotent stem cells with self-renewal and multilineage differentiation potential. Their use has been reported to be a promising therapeutic strategy for tissue regeneration in regenerative medicine [14]. Stem cells are found in nearly all adult tissues. MSCs with different origins have been reported to be used for promoting tendon-to-bone junction repair [15–21]. However, the transplantation of MSCs also has risks that may cause immune rejection, bear the potential of tumorigenicity, and result in cancer drug resistance [22]. Therefore, the further development of stem cell therapy encounters great difficulties. Recently, a large body of evidence has shown that the positive effects of stem cell therapy are likely mediated by the paracrine mechanisms of MSCs, particularly by secreting exosomes [9, 23]. Mesenchymal stem cell-derived exosomes (MSC-exos), nano-sized extracellular vesicles, have been identified as emerging nano-scale acellular therapeutic agents for tissue regeneration. They have been employed in cutaneous wound healing, ischemic brain injury, renal injuries, osteoarthritis, fracture healing, and degenerative bone disease in recent years [24–28]. Thus, cell-free therapy is anticipated to provide a new perspective for researchers in promoting tendon–bone healing.

Clinically, traditional conservative management or operative treatment is difficult to reconstruct anatomic continuity after TBI injuries. Hence, a better understanding of the mechanisms of exosomes has important implications for the design of novel therapeutic scenarios for TBI injuries. In this paper, we review the potential role of MSC-exos and the mechanisms underlying these actions in the improvement of tendon–bone healing. In addition, we discuss the possible application of MSC-exos for promoting of tendon–bone healing. Finally, the current challenges and future research directions of MSC-exos-based strategies for improving tendon–bone healing are proposed.

## Tendon–bone healing

### Physical structure of a tendon–bone insertion

**Fig. 1:**
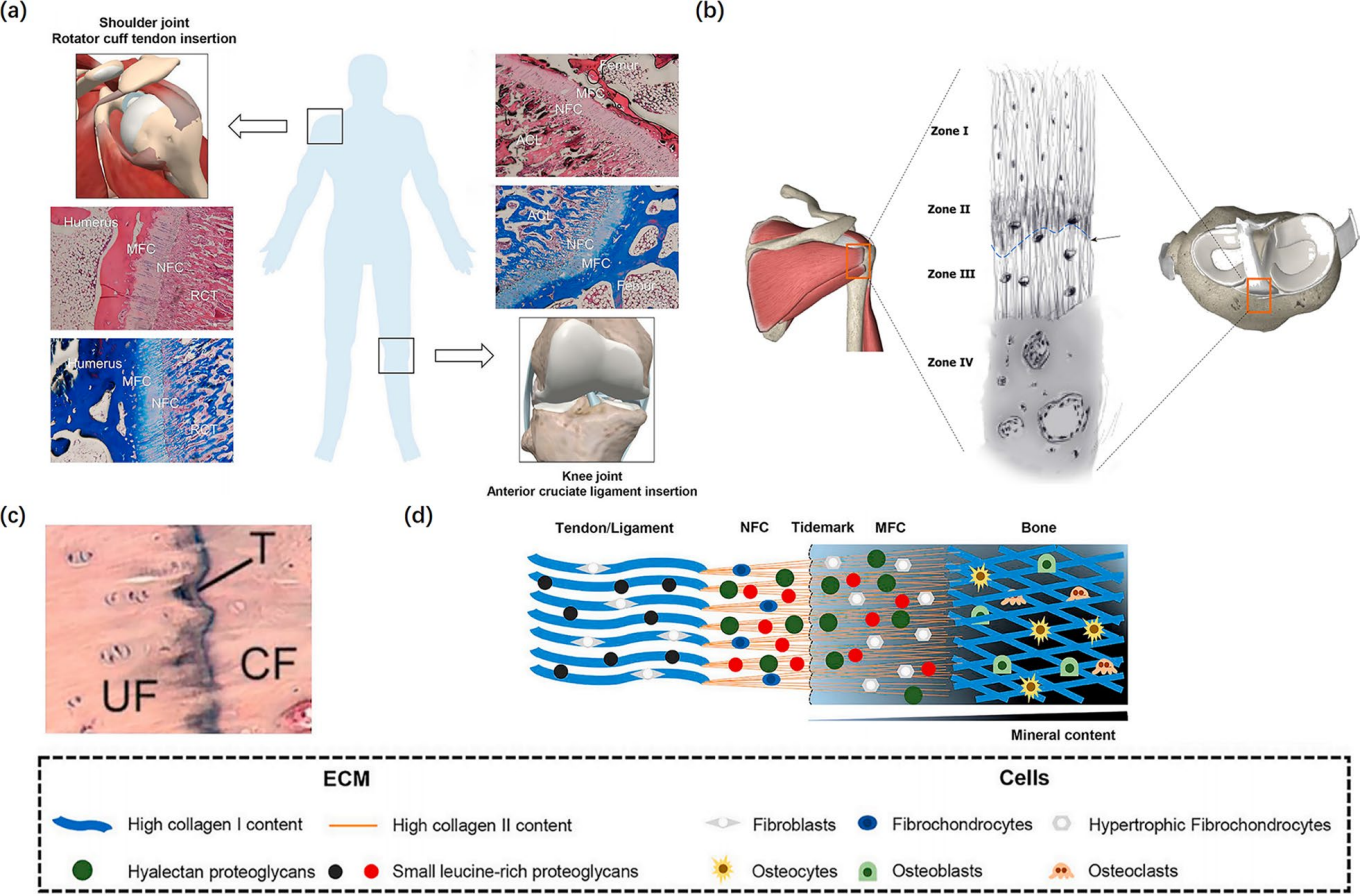
Physical Structure of tendon-bone insertion. **a** The histological staining of tendon/ligament-to-bone insertion (H&E, and Masson staining). Reproduced with permission [46]. **b** Zone I consists of the ligament. Zone II comprises nonmineralized fibrocartilage. Zone III is composed of mineralized cartilage. Zone IV consists of bone. Tidemark between Zone II and Zone III (black arrow) is shown. Reproduced with permission [7]. **c** The tidemark stained with H&E. Reproduced with permission [46]. **d** The schematic of tendon/ligament-to-bone insertion. RCT indicates rotator cuff tendon; ACL indicates anterior cruciate ligament; NFC indicates non-mineralized fibrocartilage; MFC indicates mineralized fibrocartilage; UF indicates uncalcified fibrocartilage; CF indicates calcified fibrocartilage; T indicates tidemark; ECM indicates extracellular matrix. Reproduced with permission [46]

The musculoskeletal system functions through the coordination of multiple types of connective tissues. Among them, a highly specialized and organized tissue, called tendon–bone insertion, incorporates tendon/ligament to bone [29]. This complex transition site from hard bone to soft tendon/ligament provides a strong supporting strength to promote joint movement [30]. The intact healthy enthesis effectively facilitates the progressive transfer of complex mechanical loads through its gradient structure between the two distinct, inhomogeneous tissues: tendon/ligament and bone [31]. TBIs can be divided into two types, namely, indirect and direct insertions. For example, the medial collateral ligament runs parallel to the bone and inserts into the tibial through indirect insertions. Indirect insertion sites attach to the metaphysis and diaphysis of bone by connecting the superficial layers of the tendon/ligament to the periosteum, supported by specialized collagen fibers, called fibers, which provide anchorage between the bone and tendon/ligament. In contrast, direct insertions, such as the insertion of the ACL and RC, insert onto a bone by a gradient structure consisting of four distinct yet continuous layers in the sequence from soft to hard tissues, i.e., tendon, unmineralized fibrocartilage, mineralized fibrocartilage, and bone (Fig. 1) [5, 31–33]. The increase of tissue stiffness along the insertion site from tendon/ligament to bone may be regulated by the alignment of collagen fiber and a progressive increase in mineralization [34]. The fibrocartilage zone plays a key role in absorbing stress concentration between the tendon and bone. The demineralized region withstands excessive pressure or strain within the distal tendon, while the mineralized region protects the bone from excessive shear stress [31].

### Healing processes of tendon–bone insertion

Histologically, the native insertion site is a complex layered structure, which ensures its unique function, but once the tendon–bone junctions are injured, restoration of the anatomic structures is difficult. Based on current experimentation on animals, scholars have elucidated the chronological and morphological changes in TBI [35–37]. During surgical reconstruction, because the graft is placed and pulled through bone tunnels, the transitional tissue of the native direct insertion is not regenerated. Instead, the graft heals with the formation of fibrovascular scar tissue at the graft–tunnel interface [38]. The fibrovascular tissue is disorganized at the beginning. Subsequently, the formation of perpendicular fibers resembling the fibers of an indirect insertion begins 3 to 4 weeks after graft placement [39]. In the end, gradual bone ingrowth into this interface tissue 1 year after the operation promotes the graft attachment strength and integrates the graft into the surrounding bone [38]. Studies have demonstrated that the scar tissue exhibits weaker mechanical strength owing to the disorganized collagen fibers and the lack of the gradient of mineral distribution, leading to the increase in re-tear rate [40, 41]. Currently, there is no strategy to enhance the anatomic regeneration of the enthesis. Therefore, enhancing the functional and biological recovery of TBI is significant and urgent in regenerative medicine.

The healing process of TBI is divided into four different phases: inflammation phase, proliferation phase, remodeling phase, and maturation phase [42]. For the inflammatory phase, macrophages and neutrophils were among the first to be recruited in the healing of TBI and clearing tissue debris, which was eventually followed by fibroblast infiltration and the deposition of collagen type III, leading to the formation of the fibrovascular scar tissue at the interface between the graft and the bone tunnel [43]. During the proliferation phase, the cytokines and growth factors released by interface cells stimulate the migration, proliferation, and differentiation of stem cells, contributing to revascularization [44]. Next, during the remodeling phase, these healing cells synthesize and deposit a new extracellular matrix with bone ingrowth into the graft. Gradually, the new bone, fibrocartilage, and Sharpey's fibers are formed, which establish continuity of collagen fibers between the tendon graft and the bone [45]. Finally, cellularity and vascularity at the interface gradually decrease, and the collagen fibers are mostly parallel to each other in the maturation phase, indicating gradual restoration of the biomechanical strength of the interface.

## Current biological treatments for promoting tendon–bone healing

Most cases of tendon/ligament rupture and enthesis injuries require surgical interventions. However, insufficient osteointegration after surgical reconstruction is one of the leading causes resulting in unsatisfactory clinical outcomes [47]. In 1993, Rodeo et al. reported that the mechanical strength of the TBI was positively associated with the degree of bone ingrowth into the graft, mineralization, and tissue maturation at the interface [36]. Over the past decade, researchers have studied biological treatments meticulously for accelerating and promoting tendon–bone healing in basic science studies of orthopedics. In recent years, using growth factors/cytokines [48], platelet-rich plasma [49], physical therapies [50], cell-based therapies [51], tissue engineering [52], and a range of delivery and inducement approaches have been applied and developed boomingly.

Currently, a variety of growth factors/cytokines, including transforming growth factor-β (TGF-β), bone morphogenetic protein (BMP), and granulocyte colony-stimulating factor (G-CSF), have been applied to promote tendon graft healing within a bone tunnel in various animal model studies. For instance, improving the integration of the transplanted tendon grafts in the bone tunnel with bone morphogenetic protein-7 (BMP-7) treatment after reconstruction of the ACL has been reported in sheep models [48]. However, there are still a few limitations to the clinical application of these methods. Most importantly, these biological factors are very difficult to retain in the tendon/ligament injury site and would flow away or undergo rapid clearance [6].

Cell-based therapies using stem cells exhibit the potential to become an effective treatment alternative in tissue engineering of TBI. MSCs are a stem cell population, which have the ability to self-renew and differentiate into a wide variety of cell types, including adipocytes, osteoblasts, and chondrocytes, while showing low immunogenicity upon transplantation. In addition, MSCs are capable of secreting a large number of bioactive molecules to enhance soft and hard tissue repair and regeneration [53]. In the field of tendon–bone healing, the most commonly used stem cells have been isolated from bone marrow (BMSCs) or adipose tissue (ASCs). However, several recent studies have shown that MSCs derived from the periosteum, synovium, and tendon have been widely used for improving enthesis regeneration at the tendon–bone healing site [54–56]. Despite the promising outcomes of using cellular therapies, they have a few limitations. For instance, there is no uniform standard for the dosage and frequency of stem cell therapy to accomplish the optimal effect, which is a certain obstacle to the clinical application of stem cells [7]. Besides, the potential risks of causing tumor formation and other adverse effects may occur after the administration of stem cells [57].

In brief, these limitations have necessitated the exploration of a novel therapeutic method to meet clinical requirements and enhance tendon–bone healing.

## Therapeutic potential of MSCs’ secretory products in tendon–bone healing

In recent years, a series of studies have shown that the positive effects of stem cell therapy may not be restricted to cellular regeneration alone, but they are also mediated by a large number of bioactive molecules secreted by the paracrine action of MSCs. Stem cells can secrete growth factors, cytokines, and extracellular vesicles that can modulate the molecular component of the local environment to elicit responses from resident cells, presenting antiapoptotic, angiogenic, anti-scarring, immunomodulatory, and chemoattractant functions [58]. In regenerative medicine, secretory products of stem cells, such as secretomes and exosomes, have been tested for tendon–bone healing. Recent research has demonstrated that the efficacy of MSC-derived exosome therapies is comparable to those of stem-cell-based therapies without the potentially harmful effects of entire cell transplantations [59].

### Secretome/conditioned medium (CM)

Secretome or CM, a medium where stem cells are cultured, contains various bioactive molecules secreted by living cells [60]. When the local microenvironment is altered, the MSCs released secretome or CM into the culture media [61]. The MSCs’ secretome or CM is made up of soluble proteins, lipids, nucleic acid, extracellular vesicles, and abundant cytokines/growth factors, such as vascular endothelial growth factor (VEGF), platelet-derived growth factor (PDGF), TGF, and epidermal growth factor (EGF) [62, 63]. Secretome or CM plays a pivotal role in tissue regeneration, promoting cell proliferation, migration, and differentiation [64]. Recently, several studies have suggested the beneficial effects of secretome or CM on tendon–bone healing. Sevivas et al. demonstrated that in vitro tendon cell viability and density and in vivo biochemical and histological properties of TBI were increased significantly in the CM-treated group compared with the control group [65]. Sun et al. demonstrated that intra-articular injection of a CM of human bone marrow-derived stem cells (hBMSC-CM) could promote tendon graft–bone healing in a rat model of ACL reconstruction [66]. Chen et al. found that hBMSC-CM could enhance tendon–bone healing by regulating macrophage polarization in a rat model of RC repair [32]. In light of this, we believe that MSCs’ secretome/CM is a promising candidate clinically for tendon-bone healing. However, the exact mechanism that which MSCs’ secretome/CM has the potential to be used as a therapeutic tool has not been elucidated.

### Exosomes

**Fig. 2:**
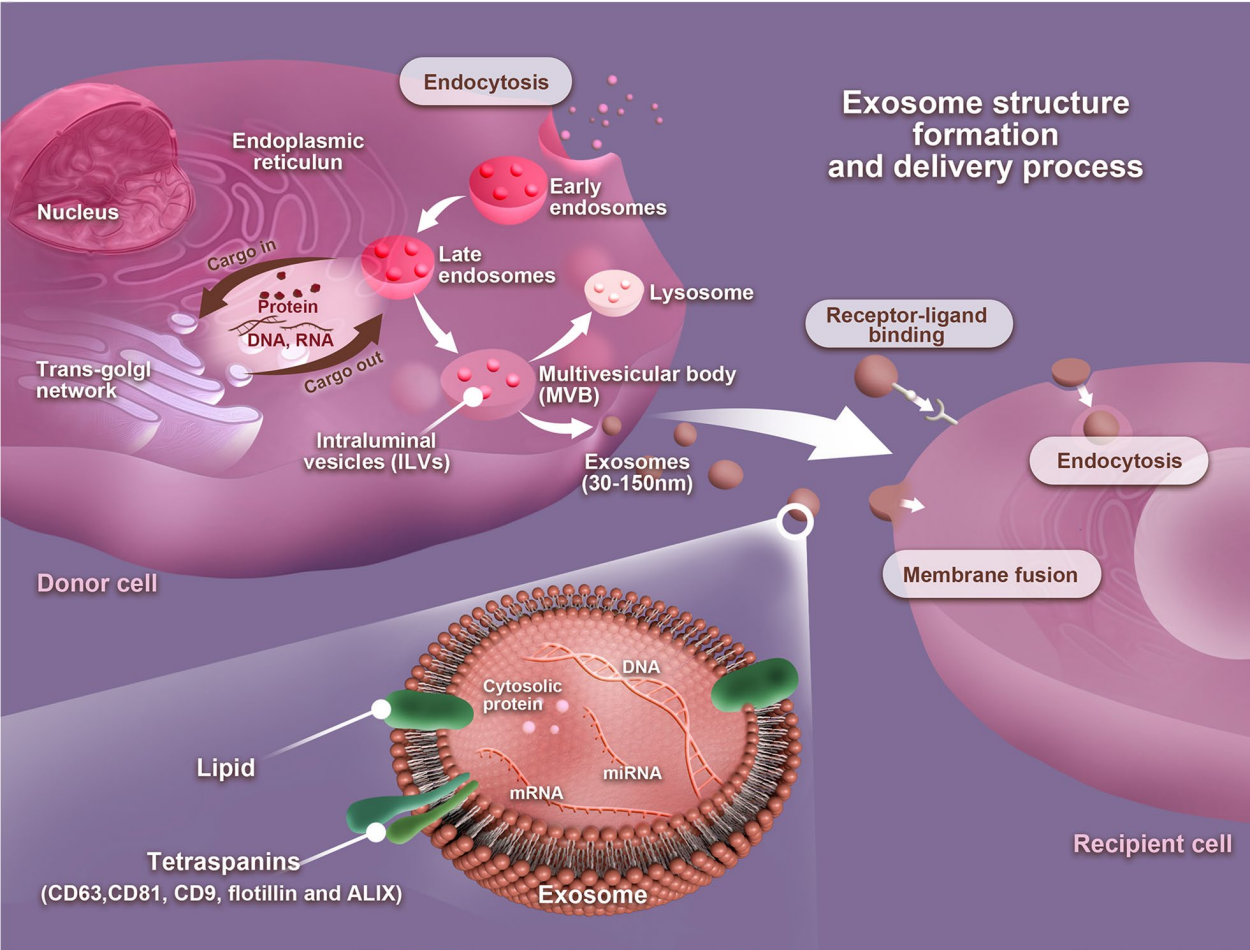
Exosome structure, formation, and delivery process. The plasma membrane invaginates to form early endosomes and then gradually forms late endosomes. Multivesicular bodies (MVBs) form by inward budding of the late endosomal limiting membrane. The MVB can either fuse with lysosomes or autophagosomes to be degraded or fuse with the plasma membrane to the release of late endosomal contents, i.e., intraluminal vesicles (ILVs) with a variety of information into the extracellular space to form exosomes. In the extracellular space, exosomes can be taken up by recipient cells via three patterns: receptor–ligand binding, endocytosis, or membrane fusion

Exosomes are a class of extracellular vesicles with a diameter ranging around 30–150 nm [67]. Exosomes are nano-sized particles with a lipid bilayer and a membrane structure that are naturally released by various cell types under both physiological and pathological conditions by cytosolic exocytosis [68]. The formation of exosomes can be divided into three different stages. Stage 1 is when the cell’s limiting membrane invaginates to form early endosomes and then gradually forms late endosomes. Stage 2 involves the inward budding of late endosomal membranes that form a multivesicular body (MVB). Stage 3 involves the fusion of an MVB with the plasma membrane that contributes to the release of late endosomal contents, i.e., intraluminal vesicles (ILVs) with a variety of information into the extracellular space to form exosomes [69, 70]. In the extracellular space, exosomes can be taken up by recipient cells via three patterns: receptor–ligand binding, endocytosis, or membrane fusion (Fig. 2) [71]. Exosomes have been widely found in cell cultures and various body fluids, and several studies have proved that exosomes play an important role in intercellular communication by transferring bioactive lipids, proteins, and nucleic acids, such as DNAs, mRNAs, miRNAs, and lncRNAs between cells to influence physiological and pathological processes of recipient cells, for instance, improving ischemic brain injury, accelerated cutaneous wound healing by enhancing angiogenesis, and therapeutic potential in osteoarthritis [26, 72, 73]. As a key secretion product of MSCs, MSC-exos are shown to have similar regenerative properties to parental MSCs. Consequently, MSC-exos have a very high potential for diagnostic or prognostic biomarkers, drug delivery systems, as well as vehicles for gene therapy in clinical applications.

## Current progress in the application of exosomes to TBI injuries

### Exosome isolation and identification

In studies of exosomes and their practical usage, the extraction and identification methods of exosomes are the research bases. First, they must be separated and purified from both biological fluids and in vitro cell cultures. The choice of exosome isolation techniques noticeably affects the exosome yield. Selection and modification of isolation techniques should vary according to the size, shape, density, and surface proteins of exosomes isolated from the biological fluid or cell culture. In this part, we briefly discuss the principles and methods of several commonly used exosome separation approaches and then summarize their respective advantages and disadvantages (Table 1) [74–79]. Additionally, the tissue source for parental MSCs for exosome studies has been isolated from a variety of tissues including bone marrow, umbilical cord, adipose tissue, embryonic stem cells (ESCs), or induced pluripotent stem cells (iPSCs). To date, the most common isolation strategy for purity exosomes from conditioned media has been ultracentrifugation and precipitation methods [80]. Unfortunately, these approaches suffer from many disadvantages, such as potential damage to exosomes and the co-precipitation of other non-exosomal contaminants like proteins [77]. Due to the limitations of current conventional methods, it is challenging to obtain the high yield, high purity, and better function of exosomes, especially MSC-exos, as the number of exosomes produced by MSCs is relatively small compared with other types of cells or body fluids. Moreover, there are homogeneity issues. Therefore, further research and development on the biological properties and extraction technologies of exosomes are urgently needed to improve and optimize the large-scale production of exosomal formulations that could meet their clinical application.

**Table 1:** Comparison of different exosome isolation methods

Exosome-based features	Isolation techniques	Equipment requirements	Time	Specificity	Pros and cons	Refs.
Density	Ultracentrifugation	Normal	Long	Yes/no	Standard gold method, low cost but the potential damage to exosomes	[76, 79, 83]
Size	Size-exclusion chromatography	High	Short	No	High concentration but low sample utilization	[77, 83]
Surface proteins	Polymer precipitation	Low	Short	No	High concentration but low purity and low sample utilization	[76, 78]
	Immunoaffinity capture	Low	Short	Yes	High concentration but low purity	[75, 79, 83]
	Microfluidics isolation	High	Short	Yes	High purity but expensive	[74, 83]

Next, it is also essential to identify the extracted exosomes for their biomedical application. According to morphological characteristics, size, and marker proteins, the identification approaches of exosomes currently primarily include nanoparticle tracking analysis, scanning electron microscopy or transmission electron microscopy observation, and marker protein expression detection as western blot analysis and flow cytometry [81, 82].

### Exosomes as nanocarriers for drug delivery system

Exosomes can be secreted by a variety of cell types, travel throughout the body by the circulatory system, and naturally cross the biological barriers and other tissues, which play an important role in intercellular communication by delivering miRNAs, mRNAs, proteins, and other bioactive substances into recipient cells [84]. At present, the native cargo delivery of exosomes has been developed as various drug delivery carriers for disease treatment, which has the advantages of no cytotoxicity, low immunogenicity, high stability in circulation, and good biocompatibility compared with cell-based therapies [85]. The cell-targeting functions of exosomes potentiate their role as drug delivery nanoplatforms. Recently, numerous studies on exosomes that act as nanocarriers have focused on miRNA molecules (Table 2). miRNAs are a class of small non-coding RNA molecules. Exosomes contain various miRNAs, which have been increasingly recognized as one of the most important regulators in disease (such as inflammation and fibrosis) and the biological processes of tissue repair and regeneration, involving angiogenesis, cellular proliferation, and differentiation by regulating the gene expression of recipient cells through translational or post-transcriptional repression [86, 87]. The key role of miRNAs in mediating exosomes to facilitate tendon–bone healing has been proven. In a seminal study, Cui et al. reported that macrophage-derived miR-21-5p-containing exosomes induced peritendinous fibrosis after tendon injury by directly targeting Smad7 in a rat model [86]. Interestingly, it has recently been found that exosomal miR-21-5p could promote fibrogenesis and enhance tendon-bone integration by reducing SMAD7 expression levels in a rat model of ACLR [88]. Furthermore, Feng et al. discovered that exosomes derived from genetically modified Scleraxis-overexpressing PDGFRα(+) BMMSCs delivered miR-6924–5p to prevent osteoclast formation during tendon–bone healing and enhance the biomechanical strength of the TBI [89]. A recent study conducted by Wu et al. found that exosomes derived from low-intensity pulsed ultrasound stimulation (LIPUS)-preconditioned bone marrow mesenchymal stem cells (LIPUS-BMSC-Exos) could improve fibrocartilage regeneration at the tendon–bone interface and decrease supraspinatus fatty infiltration in a murine RC repair model by promoting BMSC chondrogenesis and anti-adipogenesis, which was primarily by delivering miR-140 [90]. Similarly, Z. Li et al. demonstrated that BMSC-derived exosomes (BMSC-Exos) overexpressing miR-23a-3p could promote M1 macrophage to M2 macrophage polarization, reduce the early inflammatory reaction at the tendon–bone interface, and promote early healing after ACL reconstruction [91]. However, currently, most data on miRNA-mediated mechanisms of tendon–bone healing are based on the use of unpackaged miRNAs, while little is known about the effects of miRNAs existing as cargo of MSC-exos, which hinders the development of exos-derived miRNAs into clinical practices. Thus, further exploration of the mechanisms of targeted delivery of exosomal miRNAs is required, which could offer a therapeutic strategy for tendon–bone healing. Additionally, which miRNA can perform the function of exosomes should be identified. However, a recent study has shown that MSC-derived exosomes may play a functional role due to their proteins rather than miRNAs. The study demonstrated that BMSC-Exos of polyaspartic acid polylactic acid-glycolic acid (PASP-PLGA) microcapsules could facilitate tendon–bone healing by delivering bone morphogenetic protein-2 (BMP-2), which belongs to the family of transforming growth factors, and polylactic acid (PLA) [92]. This is a new point of view, like miRNAs in MSC-derived exosomes, the MSC-derived exosomes proteome plays a pivotal role in many biological processes and has the potential to modulate various biological functions related to disease pathogenesis, tissue repair, and regeneration, which may provide a new direction for researchers in enhancing tendon–bone healing.

**Table 2:** Summary of exosomal miRNAs involved in tendon-bone healing

miRNAs	Stem cell source	Models	Biological functions in tendon–bone healing	Refs.
miR-21-5p	Magnetically actuated human BMSCs	A rat model of ACLR	Significantly prevented peri-tunnel bone loss, promoted more osseous ingrowth into the tendon graft, increased fibrocartilage formation at the tendon-bone tunnel interface, promoted ECM production and tissue fibrosis in tendon-bone healing	[88]
miR-23a-3p	Genetically modified Scleraxis-overexpressing PDGFRα(+) BMSCs	A mouse model of the anterior cruciate ligament tendon-bone healing	Dramatically reduced osteoclast formation and improved tendon-bone healing strength	[89]
miR-140	Low-intensity pulsed ultrasound stimulation-preconditioned mice BMSCs	A murine rotator cuff repair model	Promoted tendon-bone interface fibrocartilage regeneration and ameliorated supraspinatus fatty infiltration	[90]
miR-23a-3p	Rat BMSCs	A rat model of ACLR	Promoted M1 macrophage to M2 macrophage polarization, reduced the early inflammatory reaction at the tendon-bone interface and promoted early healing after ACLR	[91]

Furthermore, the crux to using exosomes as drug delivery carriers for enhancing tendon-bone healing is the effective loading of the small therapeutic molecules (siRNAs, miRNAs, mRNA, and even proteins) into exosomes. Currently, there are several methods to load drug moieties into exosomes, including sonication, incubation, transfection, electroporation, extrusion, freeze–thaw cycles, surface treatments, hypotonic dialysis, and the pH gradient method [93]. The electroporation method is the gold standard, however, it influences exosome membrane stability and integrity, which reduces drug loading efficiency [94]. Another commonly used method is to incubate exosomes with the cargo for a period of time at a specific temperature. The method is simple to operate, inexpensive, and has been widely applied. But the packaging rate is relatively low [95]. These methods have their advantages and drawbacks. Thus, in-depth research on drug loading approaches will significantly promote the therapeutic effect on TBI injuries.

### Exosomes combined with biomaterials as therapeutics

**Fig. 3:**
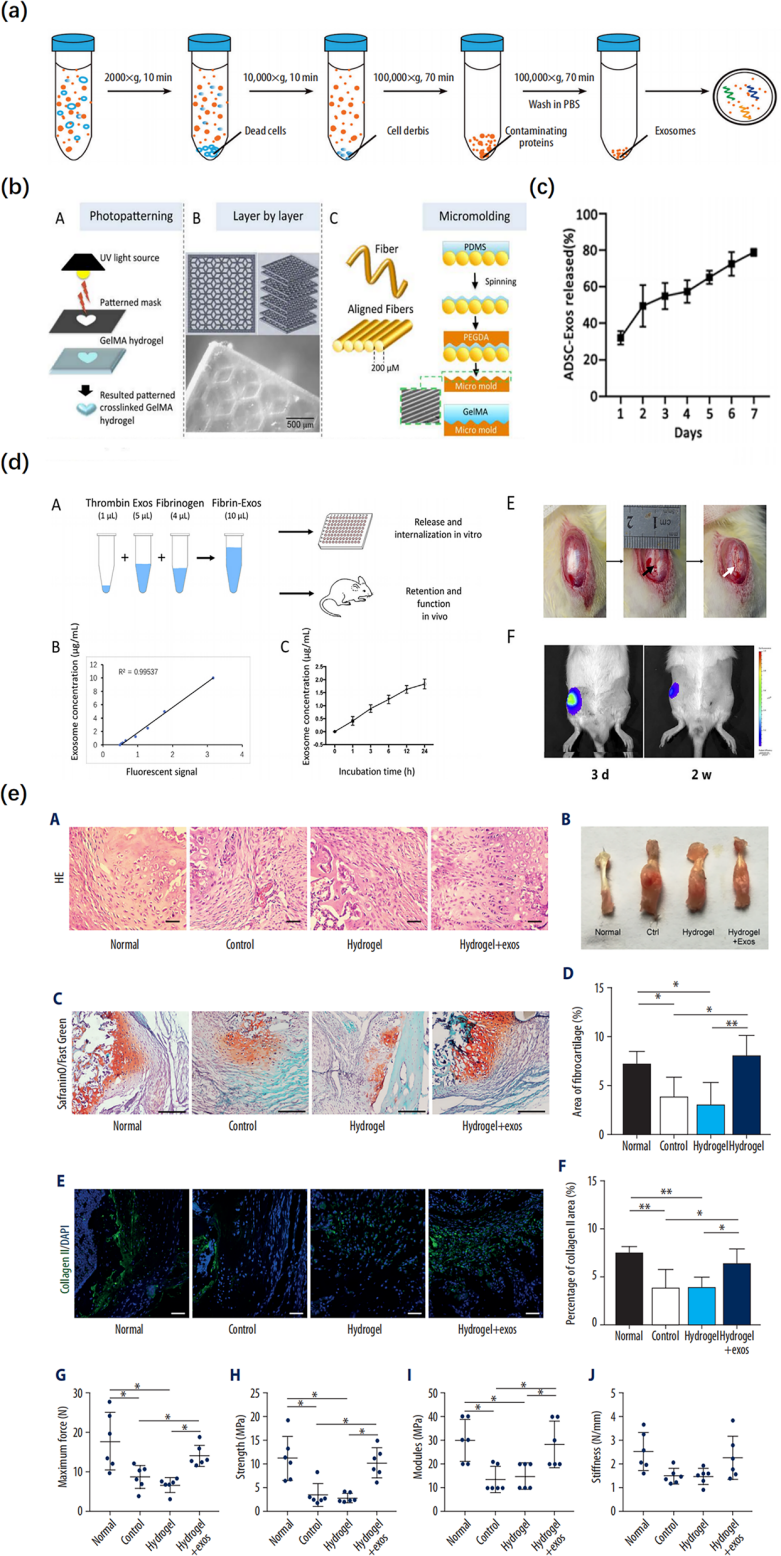
Exosomes combined with hydrogel promoted tendon-bone healing. **a** Schematic diagram of the isolation of exosomes. Reproduced with permission [105]. **b** Schematic representation of photopatterning of GelMA using a pre-patterned photomask. Stacked layers of patterned GelMA hydrogels fabricated using a micro-mirror projection stereolithography system. Schematic representation of a fiber-assisted micromolding technique for the production of parallel microgrooved surfaces that serve as a template for micropatterning GelMA. Reproduced with permission [106]. **c** Profile of ADSC-Exos released from the GelMA. Reproduced with permission [12]. **d** Release in vitro and retention in vivo of the BMSCs-exos embedded in fibrin-exo. The black arrow indicates the patellar window defect; The white arrow indicates the implanted fibrin-exo/fibrin-vesicle. Reproduced with permission [97]. **e** BMSC-Exos combined with hydrogel promoted fibrocartilage regeneration and enhanced the biomechanical properties in tendon-bone healing (H&E, safranin O-fast green, DAPI, and collagen type II staining). Reproduced with permission [105]

MSC-exos can be used directly, alone, or in combination with biomaterials to facilitate targeting and therapeutic efficacy. Typically, the application of exosomes involves the direct injection of exosome aqueous solution into the circulation system or body cavities to enhance tissue repair [12]. However, it may not be feasible to directly inject exosomes into the injury region as the free exosomes in an aqueous solution are difficult to retain in the targeted area, resulting in flowing away or undergoing rapid clearance, which contributes to exosomes being unable to exert their full biological function [96]. Therefore, a vehicle of exosomes is needed for injury repair, and it should have the properties of the controlled release of exosomes in a dose- and time-dependent manner, no adverse effects for internalization of exosomes, and a suitable degradation rate [97]. Research has evidenced that exosomes and biomaterials can be combined to promote efficacy. Currently, the most commonly used biomaterials are the following: degradable implants, tissue-derived materials, and growth factors. Furthermore, varieties of biomaterial scaffolds have been explored to deliver bioactive factors and cellular products in a controlled manner. Ideal scaffold biomaterials should have the following characteristics in interface tissue engineering to be able to promote the regeneration of the TBI: (1) the ability to reproduce the specific structure of the TBI as much as possible, including matrix composition, microstructure, and mechanical properties; (2) the scaffold can support the adhesion, proliferation, and differentiation of specific phenotypes of different types of stem or progenitor cells; and (3) the scaffold should be degradable, and the degradation rate should be comparable to the rate of tissue regeneration, which can continue to maintain the physiological load [10]. Recent studies have suggested that biomaterial scaffolds have been effectively used in tendon tissue engineering to serve as delivery vehicles for cells and growth factors, offer proper structural support, and even biochemical cues to enhance the healing and mechanical properties of the repaired tendons, indicating the possibilities of synchronous exosome delivery and tendon tissue engineering [98, 99]. In addition, hydrogels are polymer networks with high water content, which is a biologically derived and FDA-approved product with similar properties to the extracellular matrix, including excellent biocompatibility, biodegradability, and beneficial characteristics for cell infiltration and adhesion. It has been widely used in various scientific studies and clinical practices [100]. There is research that shows bioactive molecules combined with hydrogels are able to retain their structure and function for a longer period, compared to their hydrogel-free administration [101]. Recently, hydrogels have been effectively used in TBI injuries as a delivery carrier of exosomes (Fig. 3). Interestingly, it not only offers a good microenvironment for exosome storage and their gradual absorption but also delivers a great number of exosomes to the target site [97, 101]. Huang et al. found that BMSC-Exos combined with hydrogels to sustain the release of exosomes could facilitate the formation of fibrocartilage in a mouse tendon–bone construction model [102]. Similarly, in a rat RC model, Fu et al. reported that ASC-Exo loaded with hydrogels markedly enhanced osteogenic tenogenic and chondrogenic differentiation of tendon stem cells [103]. Consistent with these findings, a recent study has indicated that infrapatellar fat pad (IPFP) MSC–derived exosomes loaded in a sodium alginate hydrogel promoted tendon–bone healing and intra-articular graft remodeling in a rat ACL construction model [104]. This may provide a new therapeutic option for the application of exosomes.

Tendon–bone healing is a highly complex process. Thus, further exploration is required into the application of exosomes in tendon–bone healing to acquire a better understanding of the possible implications of exosomes for future therapies. In addition, whether exosomes need to be used for a fixed period remains to be further studied.

## Mechanisms of MSC-exos on tendon–bone healing

**Fig. 4:**
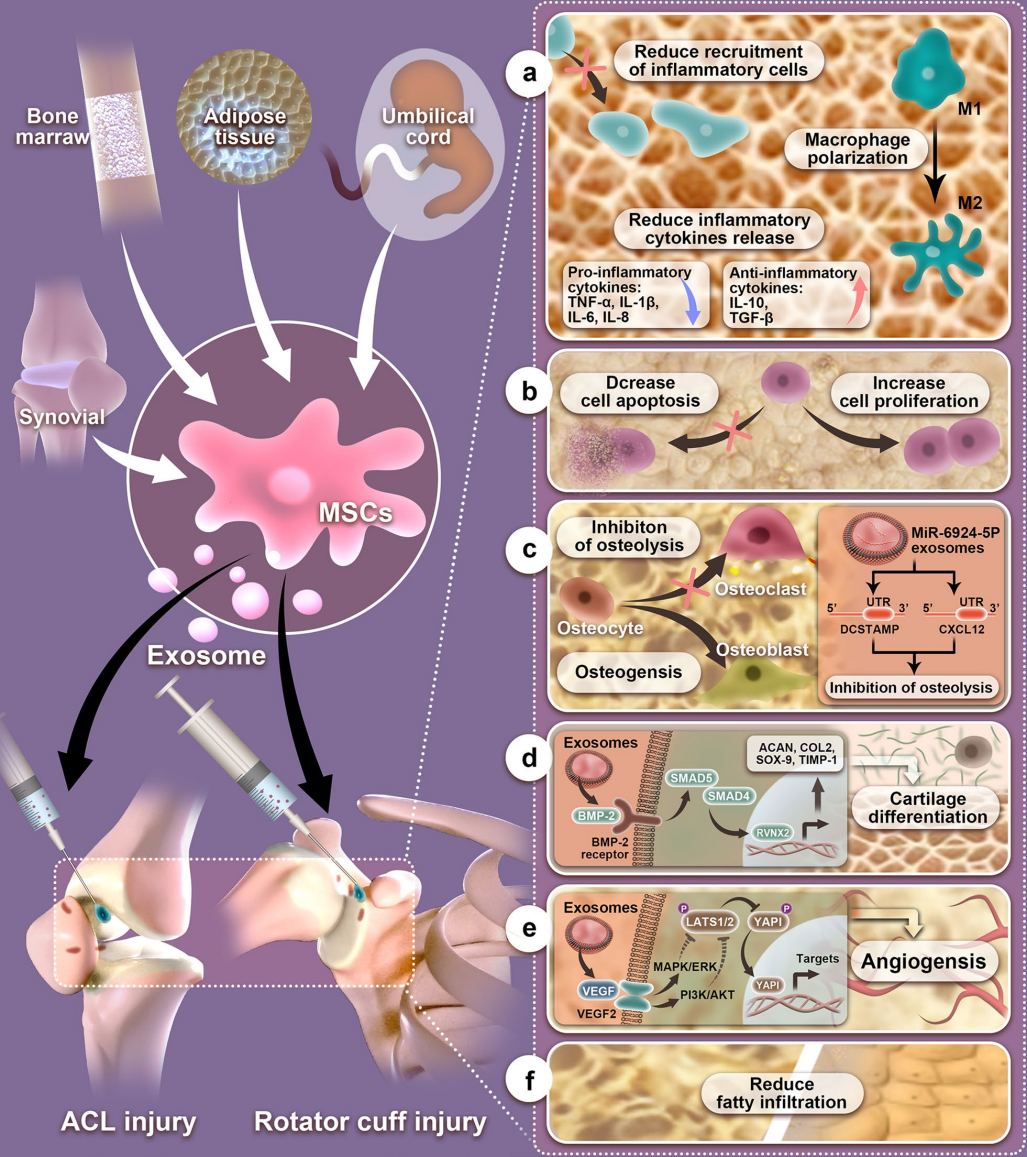
Mechanisms of exosomes on TBI injuries. **a** Exosomes could attenuate early inflammatory response via reducing inflammatory, polarizing macrophage from a pro-inflammatory M1 phenotype to a pro-regenerative M2 phenotype, and reducing inflammatory cytokines release. **b** Exosomes could enhance cell proliferation and reduce apoptosis. **c** Exosomes rich in miR-6924-5p could directly inhibit osteoclast formation by binding to the 3′-untranslated regions (3′ UTRs) of OCSTAMP and CXCL12. **d** Exosome-delivered BMP-2 promotes cartilage differentiation via Smad/RUNX2 signaling pathway. Activation of BMP-2/Smad signaling induces Runx2 expression through the activation of Smad4 and Smad5, after which Runx2 induces the expression of cartilage differentiation-related proteins, Aggrecan, Collagen II, SOX-9, and TIMP-1. **e** Exosomes promote angiogenesis by activating the VEGF and Hippo signaling pathways. When VEGF binds to the vascular endothelial growth factor receptor (VEGFR), the VEGF‐VEGFR interaction inhibits LATS1/2 and YAP1 phosphorylation by activating the PI3K and MAPK signaling pathways. The leads to increased expression of YAP1 in the nucleus inducing angiogenesis. **f** Exosomes could reduce fatty infiltration

Tendon–bone healing is a complex repair and healing process due to various reasons. First, the fibrocartilage zone has relative avascularity at the injury site, leading to slow or nonunion of the TBI; Second, once this structure is damaged, as the injury heals, fibrovascular scar tissue forms in the TBI in general, rather than through the regeneration of the fibrocartilage junction, which results in poor biomechanical properties. Third, osteointegration typically recovers slowly, leading to impairing the pullout strength and stiffness of the TBI [107–109]. Recent research has revealed the potential therapeutic benefits of using MSC-exos for TBI injuries. A better understanding of the mechanisms underlying the actions of MSC-exos on the promotion of tendon–bone healing would facilitate the development of MSC-exos as a new therapeutic strategy for the treatment of TBI injuries. The mechanisms of MSC-exos for improving tendon–bone healing are primarily ascribed to three aspects: (1) the anti-inflammatory effects of MSC-exos can reduce the infiltration of inflammatory cells into the TBI, such as macrophages, and induce macrophage polarization from the M1 phenotype to the M2 phenotype, which can reduce the pro-inflammatory cytokines, decrease cell apoptosis, increase cell proliferation, decrease the formation of fibrovascular scar tissue, and enhance the regeneration of fibrocartilaginous enthesis; (2) the angiogenesis effects of MSC-exos can provide sufficient vascular invasion around the TBI, which is essential for promoting tendon–bone healing; and (3) the osteogenesis and inhibitory osteolysis effects of MSC-exos can promote the osteointegration of the tendon graft into the bone tunnel surface, which will obtain a promising tendon–bone healing outcome in the long run. The major mechanisms that explain the rationale behind using exosomes for TBI injuries are depicted in Fig. 4.

Table 3 summarizes the major mechanisms studying the therapeutic effects of MSC-exos on tendon–bone healing.

**Table 3:** MSC-exos mediated mechanisms that enhance tendon-bone healing

Animal model	Exosomes (source/dosage/frequency)	Time point	Results	The underlying mechanisms	Refs.
Mice model of the anterior cruciate ligament tendon-bone healing	Genetically modified Scleraxis-overexpressing PDGFRα( +) BMSC-derived exosomes/${10}^{10}$ particles/once	1 week, 2, and 3 weeks after surgery	Local injection of ${BMMSC}^{\mathrm{Scx}}$-exos or miR-6924–5p dramatically reduced osteoclast formation and improved tendon-bone healing strength; inhibition of miR-6924–5p expression reversed the prevention of osteoclastogenic differentiation by ${\mathrm{BMMSC}}^{\mathrm{Scx}}$-exos; delivery of miR-6924–5p efficiently inhibited the osteoclastogenesis of human monocytes	Exosomes rich in miR-6924–5p could directly inhibit osteoclast formation by binding to the 3′-untranslated regions (3′ UTRs) of OCSTAMP and CXCL12	[89]
Rat model of rotator cuff reconstruction	Rat BMSC-derived exosomes/200 μg/once	4, and 8 weeks after surgery	BMSC-Exos increased the breaking load and stiffness of the rotator cuff after reconstruction in rats, induced angiogenesis around the rotator cuff endpoint, and promoted the growth of the tendon-bone interface	Promotion of angiogenesis through the VEGF and Hippo signaling pathways; inhibition of inflammation by inhibiting M1 macrophage polarization and M1 macrophage secretion of pro-inflammatory factors	[102]
Rat model of rotator cuff injury	Human ADSC-derived exosomes/25 μl of ADSC-Exos (0.3 mg/ml) mixed with 75 μl hydrogel/once	4, and 8 weeks after surgery	The hydrogel with ADSC-exos significantly improved the osteogenic and adipogenesis differentiation and improved the biomechanical RCT healing	Upregulation of osteogenic markers (RUNX2), cartilage markers (Sox-9), and tenogenesis genes (TNC, TNMD, and Scx)	[103]
Rat model of the anterior cruciate ligament construction	Infrapatellar fat pad (IPFP) MSC–derived exosomes/0.1 ml of IPFP MSC–derived exosomes (${10}^{10}$ particles/mL) mixed with sodium alginate hydrogel (SAH)/once	2, 4, and 8 weeks after surgery	IPFP MSC–derived exosomes group showed significantly higher biomechanical properties, a thinner graft-to-bone healing interface with more fibrocartilage, greater new bone ingrowth, and significantly fewer proinflammatory M1 macrophages and larger numbers of reparative M2 macrophages than in the other groups	Decreased M1 expression and increased M2 expression by the immunomodulation of macrophage polarization	[104]
Mice model of Achilles tendon-bone reconstruction	Mice BMSC-derived exosomes/dosage not reported/once	7 days, 14 days, and 1 month after surgery	At 1 month after surgery, there was more fibrocartilage in the hydrogel + BMSC-Exos group than in the other groups. The biomechanical properties of the tendon-bone junction were significantly promoted in the hydrogel + BMSC-Exos group	Decreased the M1 macrophages, the proinflammatory factors (IL-1b and IL-6) in local tissues and cell apoptosis, increased cell proliferation, reduced ECM deposition, and suppressed excessive scar formation by regulation of the transition of macrophages from M1 to M2	[105]
Rabbit model of chronic rotator cuff tears	Human ASC-derived exosomes/dosage not reported/once	6 and 18 weeks after injury	At the end of week 18, ASC-Exos group showed significantly lower fatty infiltration, a higher histological score with more newly regenerated fibrocartilage, and greater biomechanical properties than the saline group	May reduce the infiltration of inflammatory cells (such as macrophages) into the tendon-bone interface, which can decrease the formation of fibrous scar tissue and enhance the regeneration of the normal tendon-bone insertion site	[119]

### Regulation of macrophage polarization

**Fig. 5:**
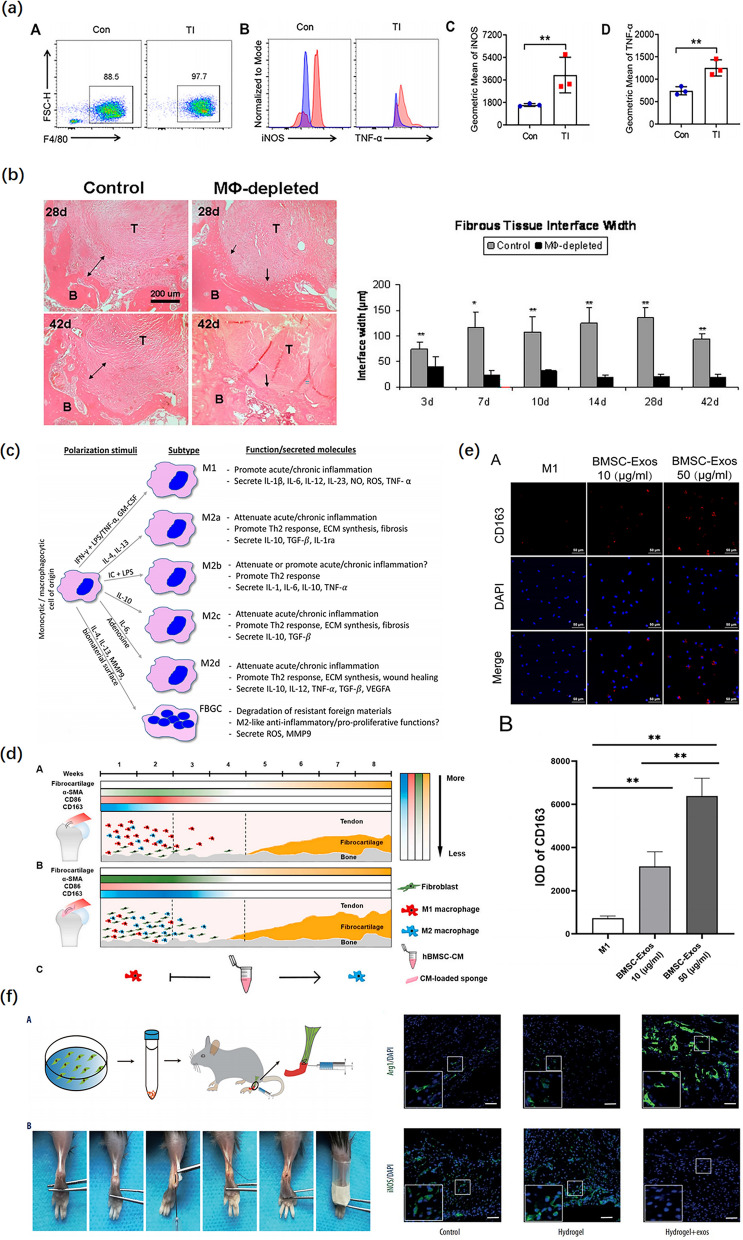
The effect of exosomes on the regulation of the transition of macrophages from M1 to M2 during tendon-bone healing. **a** An Increase in the pro-inflammatory phenotype of macrophages after a mouse tendon-bone injury (Flow cytometric analysis). Reproduced with permission [122]. **b** At all time points, the width of the fibrous tissue interface was significantly reduced following macrophage depletion (H&E staining). Reproduced with permission [112]. **c** Classification of macrophages by their inducing stimulus. Reproduced with permission [118]. **d** Brief summary of tendon-bone healing after rotator cuff repair in rats. CM indicates conditioned medium. Reproduced with permission [32]. **e** The BMSC-Exos polarized macrophages from the M1 phenotype into M2 (DAPI and CD163 staining). Reproduced with permission [91]. **f** The mouse tendon-bone reconstruction model and surgical procedure. BMSC-Exos induce M2 macrophage polarization during tendon-bone healing (DAPI, Arg1, and iNOS staining). Reproduced with permission [105]

When the graft is transplanted into bone tunnels, the earliest cellular response is the accumulation of various inflammatory cells [110]. In particular, macrophages are recruited at the graft–tunnel interface (Fig. 5a) [43]. An increasing number of studies have demonstrated that macrophages play a key role in the onset and progression of TBI injuries (Fig. 5b). Chamberlain et al. indicated that nonspecific inhibition of macrophages was detrimental to early matrix formation and ligament strength [111]. Likewise, Hays et al. reported that macrophage depletion with clodronate liposomes following ACL reconstruction resulted in significantly improved morphological and biomechanical properties at the healing tendon–bone interface, perhaps due to decreased expression of macrophage-induced TGF-β [112]. Excessive numbers of macrophages or M1 polarization can enhance cell apoptosis, reduce cell proliferation, and induce fibroblasts to secrete excessive extracellular matrix resulting in peritendinous fibrosis and the formation of scar tissue [105, 113]. Persistent inflammation leads to the release of various inflammatory mediators that impede the regeneration of fibrocartilaginous enthesis and the intra-articular graft remodeling process [114, 115].

Macrophages in areas of inflammatory tissue are derived from monocytes in the bloodstream, which are a heterogeneous group of cells having different phenotypes and functions. Activated macrophages can be categorized into two major phenotypes: first, the M1 phenotype, classically activated macrophages phenotype, participates in cellular debris clearance and host defense and accelerates the development of inflammation by secreting a large number of pro-inflammatory cytokines; second, the M2 phenotype, alternatively activated macrophage phenotype, is antiparasitic and can exert anti-inflammatory functions by releasing anti-inflammatory cytokines (such as IL-10 and TGF-β) and promote tissue regeneration and repair (Fig. 5c) [116, 117]. In the tendon–bone healing process, extensive M1 macrophage accumulation is an early event at the reconstructed place [43]. In addition, M1 macrophages can effectively polarize into M2 macrophages during the successful integration of tissue and biomaterial [118]. Consequently, regulation of the transition of macrophages from M1 to M2 attenuates the macrophage inflammatory reaction, which plays a key role in improving tendon–bone healing.

Numerous studies have shown that MSC-exos can promote tendon–bone healing by polarizing macrophages from a pro-inflammatory M1 phenotype to an anti-inflammatory M2 phenotype to reduce the inflammatory response after TBI injuries (Fig. 5d). In this regard, Wang et al. found that local injection of adipose stem cell-derived exosomes (ASC-Exos) could reduce fatty infiltration, enhance tendon–bone healing, and promote biomechanical properties in a rabbit model of a chronic RC tear, possibly due to the anti-inflammatory effects of ASC-Exos resulting in decreasing the infiltration of inflammatory cells (such as macrophages) into TBI [119]. To elucidate mechanisms underlying exosomes, Shi et al. demonstrated that the local administration of BMSC-Exos can induce M2 macrophages polarization to reduce the expression of pro-inflammatory cytokines, such as IL-1β and IL-6, as well as stimulate the expression of anti-inflammatory cytokines, such as IL-10, TGF-β, and IGF, which contributed to promoting fibrocartilage regeneration at the tendon–bone interface and improving biomechanical properties (Fig. 5f) [105]. Similarly, Huang et al. discovered that BMSC-Exos promoted tendon–bone healing after RC reconstruction in rats by inhibiting M1 macrophage polarization and M1 macrophage secretion of pro-inflammatory cytokines to suppress inflammation [102]. Consistent with these findings, a recent study has shown that IPFP MSC–derived exosomes accelerated tendon–bone healing and intra-articular graft remodeling in a rat model of ACL construction, which may have resulted from the immunomodulation of macrophage polarization, leading to decreasing M1 infiltration while promoting M2 infiltration [104]. In general, such features of exosomes make them promising tools for attenuating early inflammatory response, which is the prior condition for successful tissue healing processes [120]. However, the precise mechanism by which MSC-exos can regulate M2 macrophage polarization and function in TBI injuries has not been fully elucidated and requires further investigation.

In recent years, a growing number of studies have found that exosomes can change the polarization phenotype of macrophages by stimulating the nuclear factor-κB (NF-κB) signaling pathway. NF-κB is a key transcription factor in macrophages that regulates macrophage polarization. In a seminal study, Shen et al. demonstrated that ASC-Exos loaded onto a collagen sheet attenuated the early tendon inflammatory response, which directly targeted macrophages and subsequently reduced NF‐κB activity and downstream Il1b expression in injured tendons [121]. More interestingly, a recent study has confirmed that miR-23a-3p in BMSC-Exos enhanced the polarization of M2 macrophages and reduced the inflammatory response at the tendon–bone interface by targeting the inhibition of the IRF1 and NF-κB pathway in macrophages to promote tendon–bone healing after ACL reconstruction (Fig. 5e) [91]. Moreover, Zhou et al. reported that disulfiram (DSF), an aldehyde dehydrogenase inhibitor, significantly facilitated the transition of macrophages from M1 to M2 phenotype and decreased macrophage pro-inflammatory phenotype by targeting gasdermin D (GSDMD) to attenuate macrophage cell pyroptosis, interleukin-1β (IL-1β), and high-mobility group box 1 protein (HMGB1) release. Deficiency or inhibition of GSDMD significantly suppressed peritendinous fibrosis formation around the injured tendon and was accompanied by increased regenerated bone and fibrocartilage in tendon–bone injury. The authors observed that DSF significantly reduced the proliferation and migration of fibroblasts after BMDM exosome treatment [122]. In recent years, GSDMD with high levels of expression has been reported in the macrophages, which is involved in a variety of diseases. Heilig et al. found that GSDMD pore formation resulted in very rapid cell lysis and stimulated IL-1β secretion [123]. Therefore, NF‐κB and GSDMD may represent promising therapeutic targets for enhancing tendon–bone healing, which must be further explored.

### Regulation of angiogenesis

Tendons generally have limited vascularization when compared to muscles, especially at the TBI. After a TBI injury, poor vascularization at the tendon–bone junction decreases the availability of oxygen, growth factors, and other nutrients essential for tendon–bone healing, leading to poor biomechanical properties, eventually affecting tendon–bone healing [124]. An increasing number of studies have demonstrated that neovascularization is crucial for enhancing tendon–bone healing. Interestingly, Demirag et al. indicated that increased vascularization at the healing interface led to the more mature interface tissue that contained numerous perpendicular collagen bundles called Sharpey’s fibers [125]. Therefore, the exploration of the underlying mechanisms of angiogenesis via special pathways is critical for promoting tendon–bone healing.

**Fig. 6:**
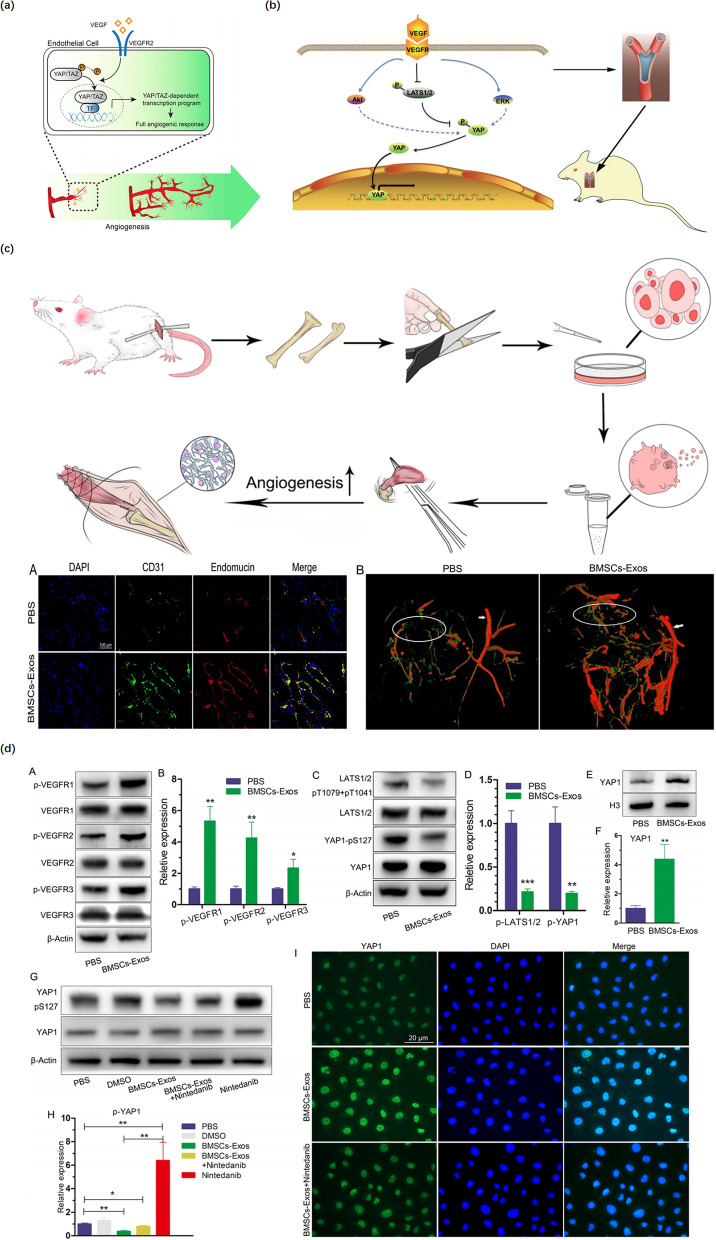
The effect of exosomes on enhancing angiogenesis. **a** YAP/TAZ orchestrate VEGF signaling during developmental angiogenesis. Reproduced with permission [126]. **b** VEGF‐induced angiogenesis via the VEGF–VEGFR–Hippo signaling pathway axis and promoted rotator tendon‐bone healing in rats. Reproduced with permission [128]. **c** BMSC-Exos promoted angiogenesis around the tendon-bone interface of the rotator cuff in rats (DAPI, CD31, endomucin staining, and angiography). Reproduced with permission [102]. **d** Functional effects of BMSC-Exos on angiogenesis-related signaling pathways in HUVECs (Western blot analysis, DAPI, and YAP1 staining). Reproduced with permission [102]

In recent years, MSC-exos have been reported to enhance tendon–bone healing, but only a few studies have investigated the effects of exosomes on tendon–bone healing associated with improving angiogenesis. In a notable study, BMSC-Exos were injected into the tail vein of rats after RC reconstruction, which improved tendon–bone healing by promoting angiogenesis through the VEGF and Hippo signaling pathways (Fig. 6c and d) [102]. Besides, Wang et al. found that YAP/TAZ, as pivotal mediators of VEGF signaling, were essential for the formation of blood vessels, and their activity was mainly regulated via the Hippo pathway [126] (Fig. 6a). VEGF is a key regulator of physiological angiogenesis that takes part in the activation, proliferation, and migration of endothelial cells in a variety of pathological processes, which can enhance blood supply in the grafted tendon in surgical reconstruction and accordingly promote tendon–bone healing (Fig. 6b) [127, 128]. Similarly, Takayama et al. discovered that suppressing VEGF expression decreased revascularization and inhibited grafted tendon maturation and biomechanical strength following ACL reconstruction surgery. Meanwhile, they also discovered that over-expression of VEGF hampered enhancement in tendon graft biomechanical strength [129]. Consequently, further studies on the mechanisms by which exosomes promote angiogenesis via the VEGF signaling pathway may provide avenues for enhancing tendon–bone healing.

### Regulation of bone metabolism

**Fig. 7:**
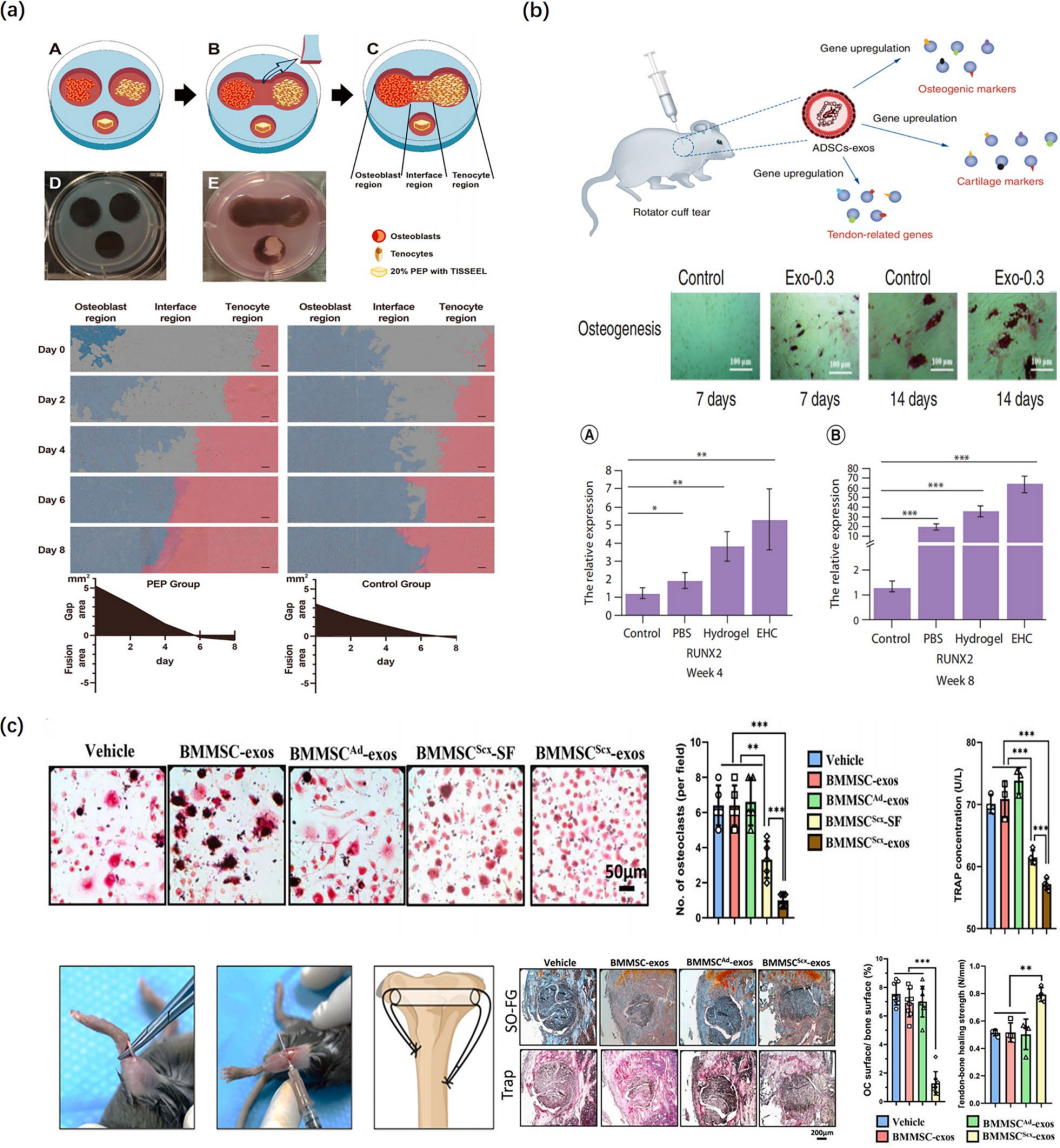
The effect of exosomes on the regulation of bone metabolism. **a** Schematic representation of the co-culture model. Cell growth and migration were significantly increased in all regions following exposure to PEP. PEP indicates purified exosome product. Reproduced with permission [73]. **b** The tendon-derived stem cell differentiation is enhanced by adipose-derived stem cell exosomes (Alizarin Red S staining). Real-time PCR analysis showed the EHC significantly induced upregulation of osteogenic marker RUNX2. EHC: Adipose-derived stem cell exosome–hydrogel complex. Reproduced with permission [103]. **c** Schematic diagram of the tendon-bone healing model. ${\mathrm{BMMSC}}^{\mathrm{Scx}}$-exos inhibit osteoclastogenesis and improve tendon-bone healing. (TRAP, and safranin O-fast green staining). ${\mathrm{BMMSC}}^{\mathrm{Scx}}$ indicates Scx-overexpressing PDGFRα (+) BMMSCs; ${\mathrm{BMMSC}}^{\mathrm{Ad}}$ indicates the control group; SF indicates soluble factors. Reproduced with permission [89]

Bone metabolism is tightly regulated by a balance of bone formation mediated by osteoblasts and bone resorption mediated by osteoclasts. Increased osteoclastic activity and reduced osteogenic activity play an important role in TBI injuries, resulting in tendon–bone healing failure after surgery. On the one hand, Rodeo et al. discovered that the tendon graft–bone interface strength positively correlated with the degree of osseous ingrowth [36]. Bone formation is a highly regulated process that involves the direct differentiation of BMSC to osteoblasts in bone remodeling in the living organism. Runt-related transcription factor-2 (RUNX2) and Osterix (Osx or Sp7), osteoblast-specific transcription factors, are essential for osteoblast differentiation and regulate many bone-related gene expressions, so-called markers of osteogenic differentiation [130, 131]. Exosomes in the TBI injury can influence osteoblast proliferation, migration, and differentiation by increasing the expression of the transcription factors RUNX2 and Osx. In this regard, Fu et al. noted that local injection of ADSC-exos-hydrogel complex in the shoulder enhanced RC repair and tendon–bone healing in a rat RC injury model, which could be because ADSC-exos induced adipogenesis and osteogenesis of tendon-derived stem cells, leading to the significant upregulation of osteogenic marker RUNX2, chondrogenic marker SOX-9, and tenogenesis genes TNC, TNMD, and Scx (Fig. 7b) [103]. On the other hand, bone resorption, i.e., osteolysis significantly impedes bony growth into the grafted tendon and decreases the pullout strength and stiffness of the TBI, resulting in delayed union and surgical failure in the early stage [132]. Tunnel osteolysis or widening after ACL reconstruction is a common complication due to increased osteoclast activity, which can occur in post-operation patients who undergo ACL reconstruction [133]. Osteoclasts are the only cell type involved in the destruction and resorption of bone tissue in vivo, which are differentiated from hematopoietic stem cell-derived monocytes and macrophage lineage progenitors (progenitors of osteoclasts). Thus, suppressing osteolysis and osteogenesis are the two central factors influencing the osteointegration of the tendon graft into the bone tunnel surface.

Numerous preclinical and clinical studies have shown the potential of using exosomes for the enhancement of tendon–bone healing via the promotion of osteogenesis and inhibition of osteolysis. In a seminal study, Feng et al. reported that MiR-6924-5p-rich exosomes derived from genetically modified Scleraxis-overexpressing PDGFRα(+) BMMSCs could efficiently suppress tunnel osteolysis and promote the biomechanical strength of tendon–bone healing via targeting two osteoclastogenic regulators OCSTAMP and CXCL12 (Fig. 7c) [89]. In another study, Ren et al. found that purified exosome products and circulating exosomes enhanced the proliferation, migration, and confluence of osteoblast cells and tenocytes in an in vitro co-culture model, especially during direct cell–cell contact, and facilitated tendon–bone healing in a rat RC tear model (Fig. 7a) [73]. Similarly, a recent study has reported that the BMSC-Exos of PASP-PLGA microcapsules via delivering bone morphogenetic protein-2 (BMP-2) and PLA facilitated the formation of bone tissue and fibrocartilage tissue as well as the stiffness and the ultimate load strength of the tendon interface by Smad/RUNX2 pathway, leading to enhancing tendon–bone healing in a rabbit model of acute RC rupture [92]. Consequently, the inhibition of osteoclast activity or osteoclastogenesis and promotion of osteogenesis provide potential targets for the enhancement of osteointegration at the TBI to facilitate tendon–bone healing.

## Challenges and future perspectives

Clinically, most patients with TBI injuries require surgical repair and reconstruction rather than nonoperative management. During the past decades, various biological strategies, such as biomaterials, stem cells, and growth factors, have been used to facilitate tendon–bone healing. However, restoring the structure of the physiological transition between tendon/ligament and bone remains a huge challenge. Poor tendon–bone healing may contribute to the re-tear of the TBI, which can dramatically reduce patients’ quality of life. In recent years, numerous studies have reported the promotional effects of MSC-exos, but these results need to be further validated in clinical research. At the same time, the clinical translation related to MSC-exos therapies still has great challenges. First, it is the absence of a gold standard protocol for the isolation of exosomes with high yield and pure product. Second, the ability, which distinguishes exosomes from other EVs, especially functional microvesicles, is scarce. Third, the optimal frequency of exosome injection is uncertain, and whether more injections could produce better results than a single injection is unclear. Extensive research must be conducted to confirm the optimal dosage and injection frequency. Fourth, exosomes can be secreted by various cell types, each with different functions; thus, further studies are required to compare exosomes derived from various cell types in clinical settings to provide the optimal choice for tendon–bone healing. Fifth, most exosome therapy trials are focused on small animal models, which have a faster rate of the tendon–bone healing process than human patients. More investigations to test the efficacy and safety of this application in large animals are necessary to prove its clinical viability. Sixth, due to the diversity and complexity of the components in exosomes, further exploration is required to determine the specific substances improving tendon–bone healing in exosomes. Lastly, further research is needed to optimize the targeting of exosomes as drug delivery vehicles, which will also resolve problems encountered by stem-cell-based therapies, and may provide a novel “all-in-one” cell-free therapeutic strategy method.

The MSC-exos research has recently become the active hotspot in the field of TBI injuries. Advancement of technologies and related research may provide more valuable information for revealing the content of MSC-exos and the underlying mechanism of MSC-exos functions. The use of exosomes as nanocarriers has a very high potential in TBI injuries. In addition, reprogrammed or redesigned exosomes for improving tendon–bone healing are promising in the future. Further studies on the mechanisms of targeted delivery of MSC-exos would clarify the engineering of MSC-exos as therapeutic carriers for drug and gene delivery, which is of great significance for the future clinical application of MSC-exos.

## Conclusion

In regenerative medicine, stem cell therapy is an effective strategy for tissue repair and regeneration. In recent years, an increasing number of studies have shown that the beneficial effects of stem cell therapy are mediated by exosomes secreted by the paracrine action of MSCs, which provide a new potential cell-free therapy for enhancing tendon–bone healing. With further research, we have found that MSC-exos may regulate macrophage polarization, promote angiogenesis, enhance osteogenesis, and suppress tunnel osteolysis. Furthermore, MSC-exos are rich in a variety of nucleic acids, multiple proteins, and bioactive lipids, which primarily function as intercellular communication carriers to deliver these between cells to trigger biological responses in recipient cells. Recent studies have shown that exosomes can combine biomaterials to facilitate tendon–bone healing. These findings may provide emerging research directions for the treatment of TBI injuries. In summary, although these studies did not establish a precise healing mechanism, they identified the great potential of exosomes in the treatment of TBI injuries.

## Data Availability

Not applicable.

## Abbreviations

TBI: Tendon–bone insertion
MSCs: Mesenchymal stem cells
EVs: Extracellular vesicles
ACL: Anterior cruciate ligament
RC: Rotator cuff
MSCs-exos: Mesenchymal stem cells-derived exosomes
RCT: Rotator cuff tendon
NFC: Non-mineralized fibrocartilage
MFC: Mineralized fibrocartilage
UF: Uncalcified fibrocartilage
CF: Calcified fibrocartilage
T: Tidemark
ECM: Extracellular matrix
TGF-β: Transforming growth factor-β
BMP: Bone morphogenetic protein
G-CSF: Granulocyte colony-stimulating factor
BMSCs: Bone marrow
ASCs: Adipose tissue
CM: Conditioned medium
VEGF: Vascular endothelial growth factor
PDGF: Platelet-derived growth factor
EGF: Epidermal growth factor
hBMSC-CM: Human bone marrow-derived stem cells
MVB: Multivesicular body
ILVs: Intraluminal vesicles
ESCs: Embryonic stem cells
iPSCs: Induced pluripotent stem cells
LIPUS: Low-intensity pulsed ultrasound stimulation
BMSC-Exos: BMSC-derived exosomes
PASP-PLGA: Polyaspartic acid polylactic acid-glycolic acid
PLA: Polyaspartic acid
IPFP: Infrapatellar fat pad
3′ UTRs: 3′-Untranslated regions
VEGFR: Vascular endothelial growth factor receptor
ASC-Exos: Adipose stem cell-derived exosomes
NF-κB: Nuclear factor-κB
DSF: Disulfiram
GSDMD: Gasdermin D
IL-1β: Interleukin-1β
HMGB1: High-mobility group box 1 protein
RUNX2: Runt-related
PEP: Purified exosome product
${\mathrm{BMMSC}}^{\mathrm{Scx}}$: Scx-overexpressing PDGFRα (+) BMMSCs
SF: Soluble factors
